# Supplementary material for: Application of seasonal-adjusted hybrid models for forecasting Discomfort Index in a heat-prone region of Bangladesh
Source: PLoS One. 2026 Mar 18;21(3):e0344556. doi: 10.1371/journal.pone.0344556 (PMC12998879; doi:10.1371/journal.pone.0344556)
Supplement: S1 File — (DOCX) [file pone.0344556.s001.docx]

**Supplementary Tables:**

**Table S1:** Forecasting performance of hybrid models for daily Discomfort Index (DI) without seasonal adjustment in Rajshahi (1985–2024).

| **Model** | **MAE** | **MAPE** | **RMSE** | **MASE** |
| --- | --- | --- | --- | --- |
| ARIMA-ANN | 4.1549 | 18.5020 | 4.6563 | 5.7395 |
| ARIMA-FP | 3.9695 | 18.0607 | 4.5127 | 5.4834 |
| ARIMA-SVR | 4.9566 | 21.3378 | 5.8507 | 6.8470 |
| ARIMA-RFR | 0.7164 | 3.1464 | 0.9437 | 0.9897 |
| ARIMA-DTR | 0.7082 | 3.1184 | 0.9447 | 0.9782 |
| ARIMA-XGBoost | 4.0192 | 18.2882 | 4.5719 | 5.5520 |
| ARIMA-LSTM | 0.7088 | 3.1198 | 0.9451 | 0.9791 |
| ARIMA-GRU | 0.7126 | 3.1289 | 0.9466 | 0.9843 |
| TBATS-ANN | 1.0677 | 4.7200 | 1.3482 | 1.4749 |
| TBATS-FP | 1.0653 | 4.7055 | 1.3423 | 1.4716 |
| TBATS-SVR | 1.0121 | 4.5107 | 1.3206 | 1.3981 |
| TBATS-RFR | 0.6821 | 3.0121 | 0.9212 | 0.9423 |
| TBATS-DTR | 0.6709 | 2.9508 | 0.8940 | 0.9268 |
| TBATS- XGBoost | 1.0666 | 4.7158 | 1.3577 | 1.4734 |
| TBATS-LSTM | 0.6712 | 2.9515 | 0.8946 | 0.9272 |
| TBATS-GRU | 0.6750 | 2.9632 | 0.8956 | 0.9324 |
| ETS-ANN | 5.5998 | 22.1143 | 6.3352 | 7.7355 |
| ETS-FP | 5.4403 | 21.6370 | 6.1358 | 7.5152 |
| ETS-SVR | 4.8749 | 19.4797 | 5.6637 | 6.7341 |
| ETS-RFR | 0.7326 | 3.2136 | 0.9599 | 1.0120 |
| ETS-DTR | 0.7196 | 3.1741 | 0.9617 | 0.9940 |
| ETS- XGBoost | 5.4588 | 21.7342 | 6.1664 | 7.5408 |
| ETS-LSTM | 0.7273 | 3.2050 | 0.9735 | 1.0046 |
| ETS-GRU | 0.7288 | 3.2164 | 0.9778 | 1.0068 |
| GARCH-ANN | 5.5215 | 21.8793 | 6.2359 | 7.6273 |
| GARCH-FP | 5.2563 | 21.0300 | 5.9173 | 7.2610 |
| GARCH-SVR | 5.0267 | 20.1611 | 5.7598 | 6.9438 |
| GARCH-RFR | 8.0749 | 33.6627 | 8.2611 | 11.1546 |
| GARCH-DTR | 8.0746 | 33.6616 | 8.2613 | 11.1542 |
| GARCH- XGBoost | 4.9457 | 20.2477 | 5.5210 | 6.8319 |
| GARCH-LSTM | 7.3055 | 30.3640 | 7.5128 | 10.0917 |
| GARCH-GRU | 8.0803 | 33.6880 | 8.2681 | 11.1620 |
| ANN-ARIMA | 3.6676 | 17.0398 | 4.8501 | 5.0663 |
| ANN-TBATS | 3.6010 | 16.7597 | 4.7886 | 4.9744 |
| ANN-ETS | 3.6666 | 17.0385 | 4.8501 | 5.0650 |
| ANN-GARCH | 5.9990 | 27.3011 | 6.8491 | 8.2869 |
| FP-ARIMA | 1.1321 | 4.9258 | 1.3997 | 1.5638 |
| FP-TBATS | 1.1733 | 5.0653 | 1.4401 | 1.6208 |
| FP-ETS | 3.5440 | 15.7635 | 3.7814 | 4.8956 |
| FP-GARCH | 2.4076 | 10.3735 | 2.7291 | 3.3258 |
| SVR-ARIMA | 0.7179 | 3.1489 | 0.9542 | 0.9917 |
| SVR-TBATS | 0.7209 | 3.1568 | 0.9555 | 0.9958 |
| SVR-ETS | 0.7179 | 3.1498 | 0.9542 | 0.9917 |
| SVR-GARCH | 4.5745 | 18.6249 | 5.0089 | 6.3192 |
| RFR-ARIMA | 0.7211 | 3.1547 | 0.9420 | 0.9962 |
| RFR-TBATS | 0.7199 | 3.1511 | 0.9413 | 0.9945 |
| RFR-ETS | 0.7211 | 3.1547 | 0.9422 | 0.9962 |
| RFR-GARCH | 4.2881 | 17.4474 | 4.7494 | 5.9235 |
| DTR-ARIMA | 1.1500 | 4.8657 | 1.3942 | 1.5885 |
| DTR-TBATS | 1.0774 | 4.6517 | 1.3290 | 1.4883 |
| DTR-ETS | 1.1339 | 4.8136 | 1.3787 | 1.5664 |
| DTR-GARCH | 2.5051 | 10.6927 | 2.8053 | 3.4606 |
| XGBoost-ARIMA | 0.7132 | 3.1321 | 0.9389 | 0.9852 |
| XGBoost-TBATS | 0.7108 | 3.1263 | 0.9378 | 0.9819 |
| XGBoost-ETS | 0.7121 | 3.1294 | 0.9384 | 0.9837 |
| XGBoost-GARCH | 3.7887 | 15.4607 | 4.1885 | 5.2336 |
| LSTM-ARIMA | 3.9447 | 18.4718 | 4.6251 | 5.4491 |
| LSTM-TBATS | 9.4106 | 36.1706 | 10.4387 | 12.9997 |
| LSTM-ETS | 5.2621 | 21.1837 | 5.9370 | 7.2689 |
| LSTM-GARCH | 5.1836 | 26.3591 | 6.7120 | 7.1606 |
| GRU-ARIMA | 3.9582 | 18.2800 | 4.5519 | 5.4677 |
| GRU-TBATS | 9.5193 | 36.5863 | 10.5200 | 13.1498 |
| GRU-ETS | 5.8931 | 23.0206 | 6.6902 | 8.1406 |
| GRU-GARCH | 4.9035 | 25.1351 | 6.4432 | 6.7736 |

**Table S2:** Forecasting performance of hybrid models for daily Discomfort Index (DI) with seasonal adjustment in Rajshahi (1985–2024).

| **Model** | **MAE** | **MAPE** | **RMSE** | **MASE** |
| --- | --- | --- | --- | --- |
| STL-ARIMA-ANN | 2.0404 | 8.9814 | 2.2577 | 2.8185 |
| STL-ARIMA-FP | 2.0339 | 8.9561 | 2.2269 | 2.8096 |
| STL-ARIMA-SVR | 2.2089 | 9.5015 | 2.4871 | 3.0513 |
| STL-ARIMA-RFR | 0.4975 | 2.1909 | 0.6628 | 0.6872 |
| STL-ARIMA-DTR | 0.5022 | 2.2125 | 0.6671 | 0.6937 |
| STL-ARIMA-XGBoost | 2.0387 | 8.9850 | 2.2457 | 2.8163 |
| STL-ARIMA-LSTM | 0.5017 | 2.2125 | 0.6699 | 0.6930 |
| STL-ARIMA-GRU | 0.5139 | 2.2580 | 0.6786 | 0.7098 |
| STL-TBATS-ANN | 0.7924 | 3.4671 | 1.0070 | 1.0947 |
| STL-TBATS-FP | 0.7954 | 3.4743 | 1.0109 | 1.0988 |
| STL-TBATS-SVR | 1.3286 | 5.5084 | 1.7821 | 1.8353 |
| STL-TBATS-RFR | 0.4877 | 2.1510 | 0.6456 | 0.6737 |
| STL-TBATS-DTR | 0.4811 | 2.1217 | 0.6378 | 0.6646 |
| STL-TBATS-XGBoost | 0.7979 | 3.4806 | 1.0162 | 1.1022 |
| STL-TBATS-LSTM | 0.4810 | 2.1230 | 0.6381 | 0.6644 |
| STL-TBATS-GRU | 0.4828 | 2.1251 | 0.6389 | 0.6669 |
| STL-ETS-ANN | 1.8188 | 8.0339 | 2.0522 | 2.5125 |
| STL-ETS-FP | 1.8106 | 8.0030 | 2.0180 | 2.5012 |
| STL-ETS-SVR | 2.0220 | 8.7123 | 2.3168 | 2.7932 |
| STL-ETS-RFR | 0.5013 | 2.2065 | 0.6664 | 0.6924 |
| STL-ETS-DTR | 0.5147 | 2.2686 | 0.6820 | 0.7110 |
| STL-ETS-XGBoost | 1.8205 | 8.0635 | 2.0473 | 2.5148 |
| STL-ETS-LSTM | 0.5227 | 2.3033 | 0.6916 | 0.7220 |
| STL-ETS-GRU | 0.5230 | 2.3036 | 0.6912 | 0.7225 |
| STL-GARCH-ANN | 8.2621 | 35.5200 | 8.3126 | 11.4132 |
| STL-GARCH-FP | 8.2623 | 35.5210 | 8.3129 | 11.4134 |
| STL-GARCH-SVR | 8.2512 | 35.4735 | 8.3019 | 11.3981 |
| STL-GARCH-RFR | 0.9797 | 4.3015 | 1.1400 | 1.3533 |
| STL-GARCH-DTR | 0.9778 | 4.2934 | 1.1384 | 1.3507 |
| STL-GARCH-XGBoost | 8.4964 | 36.5252 | 8.5455 | 11.7367 |
| STL-GARCH-LSTM | 0.9958 | 4.3711 | 1.1553 | 1.3756 |
| STL-GARCH-GRU | 0.9650 | 4.2385 | 1.1264 | 1.3331 |
| STL-ANN-ARIMA | 1.7315 | 7.4645 | 2.0475 | 2.3918 |
| STL-ANN-TBATS | 1.6952 | 7.2274 | 2.0011 | 2.3418 |
| STL-ANN-ETS | 1.6898 | 7.2129 | 1.9913 | 2.3343 |
| STL-ANN-GARCH | 7.0500 | 27.7435 | 7.9446 | 9.7388 |
| STL-FP-ARIMA | 0.8670 | 3.7451 | 1.0838 | 1.1977 |
| STL-FP-TBATS | 0.8713 | 3.7603 | 1.0881 | 1.2035 |
| STL-FP-ETS | 1.6758 | 7.4237 | 1.8960 | 2.3149 |
| STL-FP-GARCH | 3.2314 | 12.7026 | 3.6963 | 4.4638 |
| STL-SVR-ARIMA | 3.8364 | 17.8269 | 4.3926 | 5.2995 |
| STL-SVR-TBATS | 3.8323 | 17.8287 | 4.3938 | 5.2939 |
| STL-SVR-ETS | 3.8364 | 17.8248 | 4.3920 | 5.2995 |
| STL-SVR-GARCH | 8.8000 | 34.8422 | 9.8969 | 12.1561 |
| STL-RF-ARIMA | 3.8279 | 17.7998 | 4.3786 | 5.2879 |
| STL-RF-TBATS | 3.8284 | 17.7998 | 4.3785 | 5.2885 |
| STL-RF-ETS | 3.8279 | 17.7996 | 4.3785 | 5.2878 |
| STL-RF-GARCH | 8.6878 | 34.5167 | 9.7263 | 12.0011 |
| STL-DTR-ARIMA | 3.5487 | 16.8678 | 4.1998 | 4.9022 |
| STL-DTR-TBATS | 3.6053 | 16.8968 | 4.1819 | 4.9803 |
| STL-DTR-ETS | 3.5582 | 16.8695 | 4.1950 | 4.9152 |
| STL-DTR-GARCH | 6.0407 | 24.9041 | 6.6034 | 8.3445 |
| STL-XGBoost-ARIMA | 3.8373 | 17.8230 | 4.3891 | 5.3007 |
| STL-XGBoost-TBATS | 3.8389 | 17.8233 | 4.3890 | 5.3029 |
| STL-XGBoost-ETS | 3.8374 | 17.8229 | 4.3891 | 5.3010 |
| STL-XGBoost-GARCH | 8.6564 | 34.3590 | 9.7141 | 11.9578 |
| STL-LSTM-ARIMA | 1.0101 | 4.5801 | 1.2994 | 1.3954 |
| STL-LSTM-TBATS | 10.4280 | 44.4253 | 10.4931 | 14.4051 |
| STL-LSTM-ETS | 4.2472 | 17.9860 | 4.3728 | 5.8671 |
| STL-LSTM-GARCH | 6.4692 | 27.0811 | 6.6449 | 8.9364 |
| STL-GRU-ARIMA | 0.8940 | 3.92610 | 1.1857 | 1.2350 |
| STL-GRU-TBATS | 17.3210 | 73.9147 | 17.3870 | 23.9270 |
| STL-GRU-ETS | 4.8655 | 20.6430 | 4.9555 | 6.7212 |
| STL-GRU-GARCH | 5.2387 | 21.8415 | 5.4264 | 7.2366 |
