# Supplementary material for: Application of seasonal-adjusted hybrid models for forecasting Discomfort Index in a heat-prone region of Bangladesh
Source: PLoS One. 2026 Mar 18;21(3):e0344556. doi: 10.1371/journal.pone.0344556 (PMC12998879; doi:10.1371/journal.pone.0344556)
Supplement: Attachment — Submitted filename: COMMENTS.pdf [file pone.0344556.s003.pdf]

**Basic Reporting**

| Aspect | Comment & Suggestion |
|---|---|
| **Language and Clarity** | The manuscript is generally well-written and structured, but some technical sections (particularly the hybrid model descriptions) could be simplified for interdisciplinary readers. Minor editorial improvements are needed to enhance readability. |
| **Figures and Visuals** | Several figures are referenced but not fully integrated (e.g., “Insert Figure 1”). Final versions of all visuals should be included with consistent formatting, clear labeling, and appropriate resolution. Complex multi-panel visuals may benefit from division into sub-figures or supplementary materials. |
| **Terminology and Abbreviations** | While core concepts such as STL, DI, and hybrid models are introduced, some model acronyms (e.g., TBATS, ETS, GRU) would benefit from brief redefinitions upon first use to aid non-specialist readers. |
| **Contextual Relevance** | The literature review is comprehensive and well-situated in the context of climate change and thermal discomfort. However, the implications for public health and urban planning could be emphasized more strongly in the discussion section. |
| **Data Availability** | The Data Availability Statement mentions restricted access. Clarifying how other researchers can access the dataset (e.g., via a repository or controlled request) would meet open-data requirements. |

**Experimental Design**

| Aspect | Comment & Suggestion |
|---|---|
| **Study Area and Scope** | Focusing solely on Rajshahi provides depth but limits generalizability. Briefly discussing how the methodology could be scaled to other regions of Bangladesh would strengthen applicability. |
| **Model Selection Rationale** | The study evaluates 128 hybrid models, but there is limited theoretical explanation for why STL-TBATS-LSTM was expected to perform better. A brief justification highlighting its complementary seasonal and nonlinear modeling capabilities would improve clarity. |
| **Hyperparameter Details** | The manuscript lacks information on hyperparameter tuning for machine learning models (e.g., LSTM architecture, TBATS seasonal parameters). Including this information is crucial for reproducibility. |
| **Validation and Robustness** | While multiple error metrics (MAE, RMSE, MAPE, MASE) are used, the study would benefit from adding temporal validation strategies (e.g., walk-forward validation) or a sensitivity analysis to evaluate robustness. |
| **Uncertainty Quantification** | No confidence intervals or uncertainty estimates are provided for the forecasted DI trends. Including uncertainty ranges would enhance the reliability of the results for decision-making contexts. |

**Validity of the Findings**

| Aspect | Comment & Suggestion |
|---|---|
| **Alignment with Climate Trends** | The identified seasonal peaks (June–August) and long-term increase in discomfort are consistent with regional warming trends, supporting the validity of the findings. |
| **External Validation** | The results would be more credible if validated against external climate datasets (e.g., ERA5 reanalysis or nearby station data). This would demonstrate model robustness beyond the primary dataset. |
| **Limitations Discussion** | The manuscript does not sufficiently discuss limitations such as the assumption of stationary climate trends or potential shifts due to future mitigation efforts. Addressing these limitations would add balance. |
| **Policy and Practical Implications** | While the forecasts highlight growing thermal risks, the manuscript could provide stronger recommendations on how the findings inform public health preparedness, urban planning, and climate adaptation strategies. |
| **Generalizability** | The methodology is well-suited for Rajshahi, but its transferability to other regions with different climatic patterns is not explored. Future work should consider cross-regional validation. |
