# Supplementary material for: Application of seasonal-adjusted hybrid models for forecasting Discomfort Index in a heat-prone region of Bangladesh
Source: PLoS One. 2026 Mar 18;21(3):e0344556. doi: 10.1371/journal.pone.0344556 (PMC12998879; doi:10.1371/journal.pone.0344556)
Supplement: Attachment — Submitted filename: Response_to_ReviewersABA_DI.pdf [file pone.0344556.s006.pdf]

October 26, 2025

Manuscript ID: PONE-D-25-28698

Title: Application of Seasonal-Adjusted Hybrid Models for Forecasting Discomfort Index in a Heat-Prone Region of Bangladesh

Journal: PLOS ONE

Dear Editor,

We sincerely thank you and the reviewers for your valuable time and constructive comments on our manuscript. We are grateful for the opportunity to revise and resubmit. We have carefully addressed each concern, and the manuscript has been revised accordingly. Below we provide a detailed point-by-point response.

Best regards,

Authors

Editorial Requirements

When submitting your revision, we need you to address these additional requirements.

1. Please ensure that your manuscript meets PLOS ONE's style requirements, including those for file naming. The PLOS ONE style templates can be found at https://journals.plos.org/plosone/s/file?id=wjVg/PLOSOne_formatting_sample_main_body.pdf and https://journals.plos.org/plosone/s/file?id=ba62/PLOSOne_formatting_sample_title_authors_affiliations.pdf.

Author's Response:

Thank you for this reminder. We have carefully reviewed the provided PLOS ONE style templates and confirm that our manuscript now fully adheres to all formatting requirements, including file naming conventions, reference style, and manuscript structure.

2. Please note that PLOS ONE has specific guidelines on code sharing for submissions in which author-generated code underpins the findings in the manuscript. In these cases, all author-generated code must be made available without restrictions upon publication of the work.

Please review our guidelines at https://journals.plos.org/plosone/s/materials-and-software-sharing#loc-sharing-code and ensure that your code is shared in a way that follows best practice and facilitates reproducibility and reuse.

Author's Response:

We will share all author-generated code without restrictions in a public GitHub repository https://github.com/amrinbinteahmed/Discomfort-Index-Data-and-Code/tree/main upon manuscript acceptance. The repository will be cited in the manuscript, include a detailed README file. We confirm adherence to PLOS ONE's code sharing guidelines.

3. We note that you have indicated that there are restrictions to data sharing for this study. PLOS only allows data to be available upon request if there are legal or ethical restrictions on sharing data publicly. For more information on unacceptable data access restrictions, please see http://journals.plos.org/plosone/s/data-availability#loc-unacceptable-data-access-restrictions.

Before we proceed with your manuscript, please address the following prompts:

a) If there are ethical or legal restrictions on sharing a de-identified data set, please explain them in detail (e.g., data contain potentially identifying or sensitive patient information, data are owned by a third-party organization, etc.) and who has imposed them (e.g., a Research Ethics Committee or Institutional Review Board, etc.). Please also provide contact information for a data access committee, ethics committee, or other institutional body to which data requests may be sent.

b) If there are no restrictions, please upload the minimal anonymized data set necessary to replicate your study findings to a stable, public repository and provide us with the relevant URLs, DOIs, or accession numbers. For a list of recommended repositories, please see https://journals.plos.org/plosone/s/recommended-repositories. You also have the option of uploading the data as Supporting Information files, but we would recommend depositing data directly to a data repository if possible.

We will update your Data Availability statement on your behalf to reflect the information you provide.

Author's Response:

We have uploaded the fully anonymized dataset as a Supporting Information file accompanying this manuscript. We confirm our adherence to PLOS ONE's open data policy. The Data Availability statement has been updated accordingly.

4. In this instance it seems there may be acceptable restrictions in place that prevent the public sharing of your minimal data. However, in line with our goal of ensuring long-term data availability to all interested researchers, PLOS' Data Policy states that authors cannot be the sole named individuals responsible for ensuring data access (http://journals.plos.org/plosone/s/data-availability#loc-acceptable-data-sharing-methods).

Data requests to a non-author institutional point of contact, such as a data access or ethics committee, helps guarantee long term stability and availability of data. Providing interested researchers with a durable point of contact ensures data will be accessible even if an author changes email addresses, institutions, or becomes unavailable to answer requests.

Before we proceed with your manuscript, please also provide non-author contact information (phone/email/hyperlink) for a data access committee, ethics committee, or other institutional body to which data requests may be sent. If no institutional body is available to respond to requests for your minimal data, please consider if there any institutional representatives who did not collaborate in the study, and are not listed as authors on the manuscript, who would be able to hold the data and respond to external requests for data access? If so, please provide their contact information (i.e., email address). Please also provide details on how you will ensure persistent or long-term data storage and availability.

Author's Response:

We have uploaded the fully anonymized dataset as a Supporting Information file accompanying this manuscript.

5. We note that Figure 1 in your submission contain [map/satellite] images which may be copyrighted. All PLOS content is published under the Creative Commons Attribution License (CC BY 4.0), which means that the manuscript, images, and Supporting Information files will be freely available online, and any third party is permitted to access, download, copy, distribute, and use these materials in any way, even commercially, with proper attribution. For these reasons, we cannot publish previously copyrighted maps or satellite images created using proprietary data, such as Google software (Google Maps, Street View, and Earth). For more

information, see our copyright guidelines: http://journals.plos.org/plosone/s/licenses-and-copyright.

We require you to either (1) present written permission from the copyright holder to publish these figures specifically under the CC BY 4.0 license, or (2) remove the figures from your submission

a. You may seek permission from the original copyright holder of Figure 1 to publish the content specifically under the CC BY 4.0 license.

We recommend that you contact the original copyright holder with the Content Permission Form (http://journals.plos.org/plosone/s/file?id=7c09/content-permission-form.pdf) and the following text:

"I request permission for the open-access journal PLOS ONE to publish XXX under the Creative Commons Attribution License (CCAL) CC BY 4.0 (http://creativecommons.org/licenses/by/4.0/). Please be aware that this license allows unrestricted use and distribution, even commercially, by third parties. Please reply and provide explicit written permission to publish XXX under a CC BY license and complete the attached form."

Please upload the completed Content Permission Form or other proof of granted permissions as an "Other" file with your submission.

In the figure caption of the copyrighted figure, please include the following text: "Reprinted from [ref] under a CC BY license, with permission from [name of publisher], original copyright [original copyright year]."

b. If you are unable to obtain permission from the original copyright holder to publish these figures under the CC BY 4.0 license or if the copyright holder's requirements are incompatible with the CC BY 4.0 license, please either i) remove the figure or ii) supply a replacement figure that complies with the CC BY 4.0 license. Please check copyright information on all replacement figures and update the figure caption with source information. If applicable, please specify in the figure caption text when a figure is similar but not identical to the original image and is therefore for illustrative purposes only.

The following resources for replacing copyrighted map figures may be helpful:

USGS National Map Viewer (public domain): http://viewer.nationalmap.gov/viewer/

The Gateway to Astronaut Photography of Earth (public domain): http://eol.jsc.nasa.gov/sseop/clickmap/

Maps at the CIA (public domain): https://www.cia.gov/library/publications/the-world-factbook/index.html and https://www.cia.gov/library/publications/cia-maps-publications/index.html
NASA Earth Observatory (public domain): http://earthobservatory.nasa.gov/
Landsat: http://landsat.visibleearth.nasa.gov/
USGS EROS (Earth Resources Observatory and Science (EROS) Center) (public domain): http://eros.usgs.gov/#
Natural Earth (public domain): http://www.naturalearthdata.com/

Author's Response:
We thank the editor for this important note. We confirm that Figure 1 is an original creation by the authors and does not contain any copyrighted material. The map was created by the authors using the open-source software QGIS (version 3.30), utilizing our study data. The figure caption has been updated to include the required attribution: "The map was created using QGIS Geographic Information System, version 3.30 's-Hertogenbosch (https://qgis.org/project/visual-changelogs/visualchangelog330/)." We hereby grant permission for it to be published under the CC BY 4.0 license.

6. If the reviewer comments include a recommendation to cite specific previously published works, please review and evaluate these publications to determine whether they are relevant and should be cited. There is no requirement to cite these works unless the editor has indicated otherwise.

Author's Response:
Thank you. We have followed these instructions.

Reviewer #1 Comments

Comment 1: The introduction should more explicitly clarify the novelty of combining STL, TBATS, and LSTM in the context of forecasting thermal discomfort in Bangladesh. While the methodology is strong, the practical advantages of this hybrid combination compared to prior studies or conventional models should be more clearly positioned, especially regarding implications for public health interventions and urban planning.

Author's Response:

Thank you for your insightful comment regarding the novelty and positioning of our hybrid model combining STL, TBATS, and LSTM for forecasting thermal discomfort in Bangladesh. We have updated the introduction to explicitly highlight these novel contributions and their public health and urban planning implications, as follows "The novelty of our method lies in the strategic integration of seasonal-trend decomposition using Loess (STL) for effective seasonal decomposition, an advanced time series model (like Trigonometric Seasonality, Box-Cox Transformation, ARMA Errors, Trend, and Seasonal Components (TBATS)) for managing complex and multiple seasonal patterns, and an ML model (like LSTM) to capture nonlinear temporal dynamics. This integrated hybrid model surpasses conventional methods by delivering enhanced accuracy in forecasting thermal discomfort, specifically tailored to Bangladesh's highly variable climate. The resulting high-precision projections are not only a technical advancement but also play a crucial role in enabling proactive public health interventions, optimizing early warning systems, and guiding data-driven urban planning. Such improvements are essential for strengthening climate resilience among vulnerable populations in heat-prone regions like Rajshahi."

Comment 2: The model performance section is exhaustive but can be made more reader-friendly. Consider summarizing key findings by focusing on the top 3 to 5 performing models rather than detailing extensive metrics for all combinations. A concise comparative statement about why certain hybrid models outperformed others would improve readability without sacrificing analytical depth.

Author's Response:

Thank you for such insightful comments. We have streamlined the model performance section to focus on the top-performing models. For non-seasonally adjusted data, XGBoost, SVR, and RFR were the best among standalone models, whereas TBATS-DTR and TBATS-LSTM led hybrid models. For seasonally adjusted data, STL-TBATS-LSTM emerged as the top hybrid model, closely followed by STL-TBATS-DTR. These models benefit from combining decomposition (STL, TBATS) with nonlinear learning (LSTM, decision trees), effectively capturing both linear and nonlinear temporal and seasonal structures, supporting their superior forecasting accuracy. A complete listing of all hybrid models with or without seasonal adjustment and their performance metrics is provided in Supplementary Tables S1 and S2.

Comment 3: The discussion could better connect forecasted increases in discomfort levels to specific policy recommendations or adaptation strategies for Bangladesh's urban and health authorities. Adding a paragraph that suggests how stakeholders could use these forecasts for planning, such as early warning systems, work scheduling, or infrastructure design, would elevate the applied value of the research.

Author's Response:
We have expanded the Discussion section to provide actionable recommendations. We emphasize how forecasts can inform early warning systems, heat mitigation policies, urban planning interventions, and health advisories targeted at vulnerable populations. This contextualizes model outputs within practical climate adaptation and public health frameworks relevant to Bangladesh. Thank you for this good suggestion.

Comment 4: Consider adding more recent literature that examines the impact of seasonal variation on health outcomes, particularly for vulnerable populations (https://doi.org/10.1016/j.ijtst.2024.11.009), to enhance the relevance and contextual grounding of your work.

Author's Response:
We appreciate this suggestion and have incorporated the referenced recent study on seasonal variation and health outcomes, highlighting its relevance to our work. This strengthens the literature context in the Discussion section and supports the importance of accurate seasonal forecasting in managing heat-related health risks.

Reviewer #2 Comments

1. Basic Reporting
(i) Language and Clarity: The manuscript is generally well-written and structured, but some technical sections (particularly the hybrid model descriptions) could be simplified for interdisciplinary readers. Minor editorial improvements are needed to enhance readability.

Author's Response:
We simplified technical sections, particularly hybrid model descriptions, and made minor editorial improvements to enhance readability.

(ii) Figures and Visuals: Several figures are referenced but not fully integrated (e.g., "Insert Figure 1"). Final versions of all visuals should be included with consistent formatting, clear labeling, and appropriate resolution. Complex multi-panel visuals may benefit from division into sub-figures or supplementary materials.

Author's Response:
All figures have been finalized with consistent formatting, clear labels, and high resolution. As per PLOS ONE's submission guidelines, figures have been uploaded separately and will be automatically integrated into the reviewer PDF.

(iii) Terminology and Abbreviations: While core concepts such as STL, DI, and hybrid models are introduced, some model acronyms (e.g., TBATS, ETS, GRU) would benefit from brief redefinitions upon first use to aid non-specialist readers.

Author's Response:
All acronyms like TBATS, ETS, GRU have been redefined upon first use to aid interdisciplinary readers.

(iv) Contextual Relevance: The literature review is comprehensive and well-situated in the context of climate change and thermal discomfort. However, the implications for public health and urban planning could be emphasized more strongly in the discussion section.

Author's Response:
The Discussion now more strongly emphasizes public health and urban planning implications.

(v) Data Availability: The Data Availability Statement mentions restricted access. Clarifying how other researchers can access the dataset (e.g., via a repository or controlled request) would meet open-data requirements.

Author's Response:
The Data Availability Statement has been updated to clarify that the fully anonymized dataset has been uploaded as a Supporting Information file (S1 Data) accompanying the manuscript. All author-generated code will be made publicly available via a GitHub repository upon manuscript acceptance, in compliance with PLOS ONE's open-data policy.

2. Experimental Design

(i) Study Area and Scope: Focusing solely on Rajshahi provides depth but limits generalizability. Briefly discussing how the methodology could be scaled to other regions of Bangladesh would strengthen applicability.

Author's Response:

We added a discussion on generalizing the methodology to other Bangladeshi regions and tropical climates to enhance applicability.

(ii) Model Selection Rationale: The study evaluates 128 hybrid models, but there is limited theoretical explanation for why STL-TBATS-LSTM was expected to perform better. A brief justification highlighting its complementary seasonal and nonlinear modeling capabilities would improve clarity.

Author's Response:

A brief theoretical justification for STL-TBATS-LSTM's superior performance was added, emphasizing complementary seasonal and nonlinear modeling strengths.

(iii) Hyperparameter Details: The manuscript lacks information on hyperparameter tuning for machine learning models (e.g., LSTM architecture, TBATS seasonal parameters). Including this information is crucial for reproducibility.

Author's Response:

A new subsection has been added detailing the hyperparameter tuning procedures for all machine learning models, including SVR, RFR, DT, XGBoost, GRU, and LSTM architectures, as well as TBATS seasonal parameters. This addition enhances the reproducibility of our study. Please see **Section 2.5: Hyperparameter Tuning for ML Time Series Models.**

(iv) Validation and Robustness: While multiple error metrics (MAE, RMSE, MAPE, MASE) are used, the study would benefit from adding temporal validation strategies (e.g., walk forward validation) or a sensitivity analysis to evaluate robustness.

Author's Response:

We thank the reviewer for the valuable suggestion to incorporate temporal validation strategies and sensitivity analyses to evaluate model robustness. While we acknowledge the importance of these approaches for strengthening model assessment, the scope and resources of the present study limited our ability to implement them comprehensively. We recognize this as an important direction for future research and plan to extend our analysis accordingly to further validate and refine the hybrid forecasting models. We have added a statement to the Discussion section to acknowledge this limitation and to highlight plans for future work focused on comprehensive temporal validation and robustness assessment.

(v) Uncertainty Quantification: No confidence intervals or uncertainty estimates are provided for the forecasted DI trends. Including uncertainty ranges would enhance the reliability of the results for decision-making contexts.

Author's Response:
Uncertainty intervals and confidence bands for forecasted DI trends were added in Figure 8.b to improve result interpretability and decision-making support. Thank you.

3. Validity of the Findings
(i) Alignment with Climate Trends: The identified seasonal peaks (June–August) and long-term increase in discomfort are consistent with regional warming trends, supporting the validity of the findings.

Author's Response:
We thank the reviewer for acknowledging the alignment of our findings with broader climate trends. No changes were required.

(ii) External Validation: The results would be more credible if validated against external climate datasets (e.g., ERA5 reanalysis or nearby station data). This would demonstrate model robustness beyond the primary dataset.

Author's Response:
We appreciate this valuable suggestion. While we agree that external validation using datasets such as ERA5 or additional weather stations would further strengthen the robustness of our

findings, this was beyond the scope of the present study due to data access and resource constraints. To address this point, we have explicitly acknowledged the lack of external validation as a limitation and emphasized it as an important direction for future research.

(iii) Limitations Discussion: The manuscript does not sufficiently discuss limitations such as the assumption of stationary climate trends or potential shifts due to future mitigation efforts. Addressing these limitations would add balance.

Author’s Response:
We acknowledge this point. We have revised the Discussion to explicitly state that the STL-based models assume stable seasonal patterns, which may not fully capture potential nonlinear shifts in thermal discomfort trends due to climate change or future mitigation efforts. We also suggest this as a direction for future research, including exploring advanced decomposition methods (e.g., Empirical Mode Decomposition, robust STL, or wavelet transform) and newer deep learning architectures (e.g., Transformer models) to better handle such nonlinear patterns.

(iv) Policy and Practical Implications: While the forecasts highlight growing thermal risks, the manuscript could provide stronger recommendations on how the findings inform public health preparedness, urban planning, and climate adaptation strategies.

Author’s Response:
We have expanded the Discussion to strengthen policy and practical implications. The revised section emphasizes actionable strategies such as early warning systems, targeted public health interventions, and urban planning measures to mitigate heat stress, making the findings more directly relevant for decision-makers in Rajshahi and similar heat-prone regions.

(v) Generalizability: The methodology is well-suited for Rajshahi, but its transferability to other regions with different climatic patterns is not explored. Future work should consider cross-regional validation.

Author’s Response:
We have added a statement in the Discussion noting that while the methodology is tailored for Rajshahi, it could be extended to other regions in Bangladesh or similar tropical areas to

evaluate generalizability and cross-regional performance. This highlights the potential applicability of our approach beyond the study area and identifies future research directions.
