# Supplementary material for: Application of seasonal-adjusted hybrid models for forecasting Discomfort Index in a heat-prone region of Bangladesh
Source: PLoS One. 2026 Mar 18;21(3):e0344556. doi: 10.1371/journal.pone.0344556 (PMC12998879; doi:10.1371/journal.pone.0344556)
Supplement: Attachment — Submitted filename: Reviewer Report_R1.pdf [file pone.0344556.s005.pdf]

Reviewer Report_R1

Title: Application of Seasonal-Adjusted Hybrid Models for Forecasting Discomfort Index in a Heat-Prone Region of Bangladesh

Manuscript ID: PONE-D-25-28698R1

No.	Reviewer Comment	Status	Suggestions	Page No. (Revised Manuscript / Response File)
1	Simplify technical sections (hybrid model descriptions) for interdisciplinary readers	Addressed	No further action needed; ensure readability maintained throughout methods section	Response p.110
2	Finalize and properly integrate all figures	Addressed	Ensure final production PDF integrates figures correctly	Response p.110
3	Redefine acronyms (TBATS, ETS, GRU) at first use	Addressed	Verify consistency across full manuscript	Response p.110
4	Strengthen public health & urban planning implications	Addressed	None; expanded policy discussion is adequate	Discussion p.90–91
5	Clarify data access and open-data compliance	Addressed	Dataset uploaded as Supporting Information	Response p.3
6	Discuss scalability beyond Rajshahi	Addressed	Acceptable; empirical cross-regional validation can be future work	Response p.8
7	Provide clearer model selection rationale (why STL-TBATS-LSTM)	Addressed	Rationale clearly added in introduction	Response p.109
8	Add temporal validation (walk-forward) or sensitivity analysis	Addressed	Consider brief walk-forward validation or clarify split strategy more explicitly	Response p.37
9	Include uncertainty quantification (confidence intervals for forecasts)	Addressed	Add prediction intervals or bootstrap-based uncertainty ranges	Forecast results p.1–2
10	External validation using ERA5 or other datasets	Addressed	Acceptable for current study; future research noted	Response p.8
11	Expand limitations (stationarity, climate shifts)	Addressed	No further action required	Response p.8

12	Add more recent literature on seasonal health impacts	Addressed	Recently suggested reference incorporated	Response p.110
